# Induced Unbalanced Linguistic Ordered Weighted Average and Its Application in Multiperson Decision Making

**DOI:** 10.1155/2014/642165

**Published:** 2014-07-17

**Authors:** Lucas Marin, Aida Valls, David Isern, Antonio Moreno, José M. Merigó

**Affiliations:** ^1^Departament d'Enginyeria Informàtica i Matemàtiques, Universitat Rovira i Virgili, Avinguda Països Catalans 26, 43007 Tarragona, Catalonia, Spain; ^2^MBS Decision Solutions, Manchester Business School Decision and Cognitive Sciences Research Centre (DCSRC), The University of Manchester, Booth Street West, Manchester M15 4PB, UK

## Abstract

Linguistic variables are very useful to evaluate alternatives in decision making problems because they provide a vocabulary in natural language rather than numbers. Some aggregation operators for linguistic variables force the use of a symmetric and uniformly distributed set of terms. The need to relax these conditions has recently been posited. This paper presents the induced unbalanced linguistic ordered weighted average (IULOWA) operator. This operator can deal with a set of unbalanced linguistic terms that are represented using fuzzy sets. We propose a new order-inducing criterion based on the specificity and fuzziness of the linguistic terms. Different relevancies are given to the fuzzy values according to their uncertainty degree. To illustrate the behaviour of the precision-based IULOWA operator, we present an environmental assessment case study in which a multiperson multicriteria decision making model is applied.

## 1. Introduction

Aggregation operators aim to reduce a set of values into a single one that summarizes the inputs in a certain way [1, 2]. Aggregation is a key point in decision making and information fusion. In this paper we propose a new aggregation operator from the family of OWA operators for use with linguistic data. The ordered weighted averaging (OWA) operator is characterised by ordering the values of the arguments before they are aggregated according to a certain combination policy [3] with which the decision maker can determine the compensatory behaviour of the aggregation from high simultaneity (andness) to complete replaceability (orness).

An interesting extension of the OWA operator is the induced OWA (IOWA) operator [4]. Its main difference is that it uses a reordering process that is based on order-inducing variables. Thus, it is able to consider an additional reordering criterion that does not depend on the values of the arguments. Since it appeared, it has been studied by many authors that have developed several extensions [5–10] for group decision making problems.

**Figure 1:**
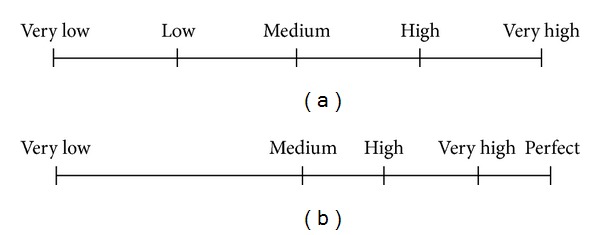
Examples of balanced (a) and unbalanced (b) linguistic term sets with five labels.

Although the OWA and IOWA operators have been traditionally applied to numerical data, we can find many applications where they have been used with linguistic variables [11]. Most of the studies in this area have assumed a uniform and symmetrical distribution of the linguistic terms that define the linguistic variable [12, 13] (see Figure 1(a)). However, there are some situations that cannot be modelled with symmetric linguistic variables [14–16]. For example, some decision making problems, such as personnel examination or project investment selection, often require a linguistic scale that assigns a different precision to each label. This issue is illustrated in the term set (b) in Figure 1, where the deviation between the indices of two adjoining labels is much larger between* very low* and* medium* than between* medium* and* high*. Some unbalanced and linguistic aggregation operators have recently appeared [2, 16–19].

This paper takes another step forward by suggesting new unbalanced linguistic aggregation operators for decision making; in particular, we define the* induced unbalanced linguistic ordered weighted averaging* (IULOWA) operator. In order to make the operator applicable in many situations, we allow the set of linguistic labels to be unbalanced. Labels can be represented by asymmetric fuzzy sets, and they can be nonuniformly distributed in the domain. Each label has an associated fuzzy set on the variable's reference scale of measurement. The fuzzy sets of the labels define a fuzzy partition. First, the ULOWA (unbalanced linguistic ordered weighting averaging) operator is presented. Unlike its predecessor, the LOWA operator [20], ULOWA is based on the extension principle and so it carries out operations on the fuzzy sets associated with the labels, thus defining a new procedure for aggregating a pair of labels according to their membership functions and the set of available linguistic terms. After this, we propose a generalization, which we call IULOWA, which enables us to work with inducing variables.

This paper shows that IULOWA fulfills the monotonicity, identity, idempotency, and boundary conditions usually required in aggregation operators. Like the other OWA operators, IULOWA provides a family of aggregation operators that is parameterized between the linguistic minimum and maximum and that includes a wide range of particular cases such as the unbalanced linguistic average (ULA), the unbalanced linguistic OWA (ULOWA), the unbalanced linguistic weighted average (ULWA), and many others. Note that, in the literature, there are approaches that employ abbreviations similar to the ones used in this paper, although they are conceptually different. In particular, it is worth mentioning that the work by Xu on uncertain linguistic variables produces the* induced uncertain linguistic OWA* (IULOWA) operator [21]. However, that approach has important differences with respect to the IULOWA operator presented in this paper. The present study is focused on the use of unbalanced linguistic information while Xu's work [21] is based on uncertain linguistic variables that deal with imprecise information when representing the linguistic labels.  Section 6 compares both approaches and highlights their similarities and differences.

Another important contribution of this paper is the proposal to use some of the information related to the definition of the labels as an order-inducing criterion in the IULOWA operator. Although the order of the arguments can be decided by taking into account the domain requirements, it is sometimes desirable to take into consideration the amount of information contained in the terms themselves. In this paper we propose a method to calculate the set of weights of the arguments taking into account the degree of uncertainty of the labels. This permits us to order the arguments by giving priority to more specific values because these represent more precise information. The method uses two well-known measures of fuzzy sets, namely, fuzziness and specificity.

We demonstrate the behaviour of the precision-based IULOWA operator with a case study in a real application. Specifically, we analyse the results obtained from the evaluation of the environmental impact produced when sewage sludge coming from wastewater treatment plants is used as fertilizer on agricultural soils. In this application, a two-stage aggregation is needed because we have a set of experts that evaluate a set of options using the same set of criteria (i.e., variables). For this reason, we further extend the IULOWA operator by using multiperson techniques in the analysis [22] and in doing so we define the multi-person-IULOWA (MP-IULOWA) operator. By including the opinions of several experts, we obtain more reliable results because we can base the decision on the knowledge of a group of people rather than on the opinion of a single individual. Moreover, the use of unbalanced and induced information enables us to deal with complex environments where some of the information is more representative and therefore needs to be prioritised in order to correctly assess the aggregation.

The rest of the paper is organised as follows.  Section 2 provides some preliminaries, introducing the OWA and IOWA operators, the linguistic OWA operator, and the management of unbalanced linguistic labels, which are the basis of the new operator.  Section 3 defines the new induced unbalanced LOWA operator, studies its properties, and presents some specific operators that can be derived from the general formulation.  Section 4 describes how the different degrees of uncertainty in the unbalanced terms can be used to induce an order among them. It also explains how the set of weights for the operator can be determined using the uncertainty measures.  Section 5 shows the application of the new IULOWA operator to a multiperson multicriteria problem (MP-IULOWA).  Section 6 compares the operator defined in this paper with the homonym IULOWA operator proposed by Xu [21]. Finally, Section 7 gives the main conclusions of the paper and suggests some lines for future research.

## 2. Preliminaries

This section describes the set of concepts required before the introduction of the IULOWA operator. First, we present numerical aggregation with the ordered weighted average (OWA), together with its order-induced extension, the induced OWA operator. We then present the LOWA (linguistic OWA) operator as the basis of the IULOWA operator proposed in this paper. Finally, we introduce recent studies on the management of unbalanced sets of terms.

### 2.1. Induced Ordered Weighted Average (IOWA) Operator

The OWA operator is formally defined as follows [3].

An OWA operator of dimension *m* is a mapping OWA  :  *R* ^*m*^ → *R* defined by an associated weighting vector *W* of dimension *m* so that ∑*w*_*i*_ = 1 and *w*_*i*_ ∈ [0,1], according to the following formula:

(1) $$\begin{array}{c} \text{OW}{\text{A}}_{W}\left(a_{1},a_{2},\ldots ,a_{m}\right)=W^{T}\cdot B=\overset{m}{\underset{j=1}{\sum }}w_{j}b_{j}, \end{array}$$

where *b*_*j*_ is the *j*th largest *a*_*i*_.

One of the main problems of the OWA operator is its dependency upon the form of the weighting vector. There are two main approaches [23, 24] to define this vector: the orness-based approach and the analytical-based approach. The first family of methods tries to optimize certain features (e.g., the variance, the maximum dispersion, and entropy) under a given orness level. The second type of methods defines the weighting method using natural language [25, 26]. These methods permit the use of absolute quantifiers such as* much more than* 10 and relative quantifiers such as* half*,* all*, and* there exists*.

Yager [25] proposed a method to obtain the OWA weighting vector by using regular increasing monotone (RIM) quantifiers. A RIM quantifier defines a fuzzy subset *Q* of the real line with (0) = 1, *Q*(1) = 1, and *Q*(*x*) ≥ *Q*(*y*) if *x* > *y*. With a RIM quantifier *Q*, the OWA weighting vector can be obtained as follows:

(2) $$\begin{array}{c} w_{i}=Q\left(\frac{i}{n}\right)-Q\left(\frac{i-1}{n}\right). \end{array}$$

For instance, the quantifier* all* is represented by the fuzzy subsets *Q*(1) = 1 and *Q*(*x*) = 0 for *x* ≠ 1.

Once the weights have been established, the aggregation policy is fully determined because the order of vector *B* in the OWA operator is based only on the value of the arguments *a*_*j*_. However, as shown by Yager and Filev [4], by allowing other orderings for the arguments we can obtain a more general aggregation operator: the IOWA (induced OWA). This generalization takes into account the ordering that an additional variable (*u*) induces in the set of values to be aggregated.

The IOWA operator is defined as follows [4]:

(3) IOWAW(〈u1,a1〉,〈u2,a2〉,…,〈um,am〉) =WT·Bu=∑j=1mwjbj,

where *W* = (*w*_1_,…, *w*_*m*_) is the usual weighting vector that defines the aggregation policy of the OWA operator, with *w*_*i*_ ∈ [0,1], ∑*w*_*i*_ = 1. The ordered argument vector *B*_*u*_ is obtained by taking *b*_*j*_ as the *a*_*i*_ value of the pair 〈*u*_*i*_, *a*_*i*_〉 which has the *j*th largest *u*_*i*_ value. Yager refers to *u* as the order-inducing variable and *a* as the argument variable.

It is important to note that the only requirement for the *u* variable is that it must be drawn from a space in which there is some linear ordering. This allows different kinds of criteria to be used for the order-inducing variables. An important aspect of the IOWA operator is the fact that the order induced by the variable *u* can produce ties in some arguments. In this case, the relative order of two arguments *a*_*i*_ and *a*_*j*_ with *u*_*i*_ = *u*_*j*_ is relevant because they may correspond to different values, that is, *a*_*i*_ ≠ *a*_*j*_. Many papers adopt the solution of replacing *a*_*i*_ and *a*_*j*_ with their arithmetic average (*a*_*i*_ + *a*_*j*_)/2. Another mechanism for solving ties consists of including a secondary ordering criterion [27], as we will propose in this paper.

The IOWA operator has the properties of monotonicity, idempotency, symmetry, homogeneity, shift-invariance, and duality [28].

The semantics of the OWA operator is a generalization of the idea of averaging or summarizing the arguments. However, IOWA permits other kinds of aggregation of the argument variables, which can be modelled by choosing the appropriate order-inducing variable. Since the introduction of the IOWA operator, several authors have proposed different ways of inducing the order. For example, Pasi and Yager [29] used IOWA to define the majority opinion in group decision making, by inducing the order of the arguments on the basis of the similarity among one value and its neighbours. The combination of this ordering criterion with linguistic quantifiers allows calculating the fulfillment of the proposition “*the satisfaction value of most of the criteria*” rather than “*most of the criteria have to be satisfied*” (which would be the result of classical OWA). So, IOWA can give different aggregation semantics. Merigó and Casanovas have developed several applications of IOWA with uncertain information [30] and with distance measures [22]. Wei et al. [31] and Xia and Xu [32] have studied several extensions by using intuitionistic fuzzy sets and fuzzy numbers.

The main advantage of the IOWA operator over the OWA operator is that it can deal with complex reordering processes where the highest value is not the first one in the reordering. Therefore, the induced variables solve an important drawback of the OWA operator, which is exclusively based on a weighting policy. For example, a journal may determine an optimal average number of pages per paper. Thus, by using the IOWA operator we can ensure that extremely long papers are not the first in the reordering process because they are not optimal in this analysis. Other interesting examples can be found when analysing several key variables of the human body including temperature, calories, and weight.

The main classes of models with induced aggregation are classified [28] as* standard auxiliary ordering*, where the inducing variable is an attribute associated with the input that is not considered in the actual aggregation process but that is informative about the object itself;* nearest-neighbour rules*, where the order-inducing variable represents the similarity or distance among the aggregating elements;* best-yesterday models*, applied in models where it is necessary to predict the order based on previous observations;* aggregation of complex objects*, in which it is necessary to operate with compound objects, such as aggregating matrices, where the order is not directly defined and needs to be estimated with some additional measure;* group decision making*, an area in which it has been proposed that the consensus can be better achieved with inducing variables based on the support of each individual score; and* multiple inducing variables*, where a priority order is established among more than one inducing variable.

### 2.2. Linguistic OWA (LOWA) Operator

In this paper we will study the aggregation of a set of linguistic terms. All the values belong to the same linguistic scale of measurement: *S* = {*s*_*i*_}, *i* ∈ {0,…, *T*}. This set *S* is defined as a finite and totally ordered term set on a reference domain *X* = [0,1], with an odd cardinal, where one of the labels corresponds to the neutral value and the remaining terms are placed around it [7, 15, 20]. The cardinality of the set must be small enough so as not to impose useless precision and rich enough in order to allow an appropriate discrimination level. The usual cardinality values are 7 or 9.

There are different approximations for working with linguistic labels. Four paradigms are distinguished [13]: operators based on the linear ordering of the labels, operators based on the extension principle, operators based on the 2-tuple model, and operators defined directly on the symbols (i.e., computing with words). The aggregation operators that follow the extension principle on linguistic data can be defined as

(4) $$\begin{array}{c} S^{n}\overset{F^{*}}{\to }F\left(R\right)\overset{\text{app}\left(\cdot \right)}{\to }S, \end{array}$$

where *S* ^*n*^ symbolizes the *n* Cartesian product of *S*, F*  is an aggregation operator based on the extension principle, *F*(*R*) is the set of fuzzy sets over the set of real numbers *R*, and app : *F*(*R*) → *S* is a linguistic approximation function that returns a label from *S* whose meaning is the closest to the obtained unlabelled fuzzy number.

Following this principle, the LOWA operator [20] is the basis of the operator that we will present in this paper. LOWA is an adaptation of the OWA operator for dealing with linguistic labels. It operates directly on the labels that are aggregated in pairs by using a convex combination operation based only on the position of the labels in the scale *S*. Because of its simplicity, it has been used in many domains [33–35].

If *A* = {*a*_1_,…, *a*_*m*_} is the set of labels to aggregate, the LOWA operator is defined as

(5) LOWA(a1,…,am)=W·BT=Cm{wk,bk,k=1,…,m}=w1⊗b1⊕(1−w1) ⊗Cm−1{βh,bh,h=2,…,m},

where *W* = (*w*_1_,…, *w*_*m*_) is an *m*-dimensional weighting vector, so that *w*_*i*_ ∈ [0,1] and ∑*w*_*i*_ = 1; *β*_*h*_ = *w*_*h*_/∑_2_^*m*^ *w*_*k*_, *h* = 2,…, *m*, and *B* is the associated ordered label vector (each element *b*_*i*_ ∈ *B* is the *i*th largest label in *A*). *C* ^*m*^ is the convex combination operator of *m* labels; if *m* = 2 it is defined as

(6) C2{wi,bi,i=1,2}=w1⊗sj⊕(1−w1)⊗si=sk,sj,si∈S(j≥i),

so that

(7) $$\begin{array}{c} k=\min\left\{T,i+\text{round}\left(w_{1}\cdot \left(j-i\right)\right)\right\}, \end{array}$$

where *b*_1_ = *s*_*j*_ and *b*_2_ = *s*_*i*_. If *w*_*j*_ = 1 and *w*_*k*_ = 0 for all *k* ≠ *j*, then *C* ^*m*^{*w*_*i*_, *b*_*i*_, *i* = 1,…, *m*} = *b*_*j*_, and* round* is the common mathematical function which translates a real number to its nearest integer value.

It is worth noting that the LOWA operator is commutative, monotonic, bounded, and idempotent. These properties will be used later to analyse our proposed IULOWA operator.

### 2.3. Management of Unbalanced Linguistic Labels

**Figure 2:**
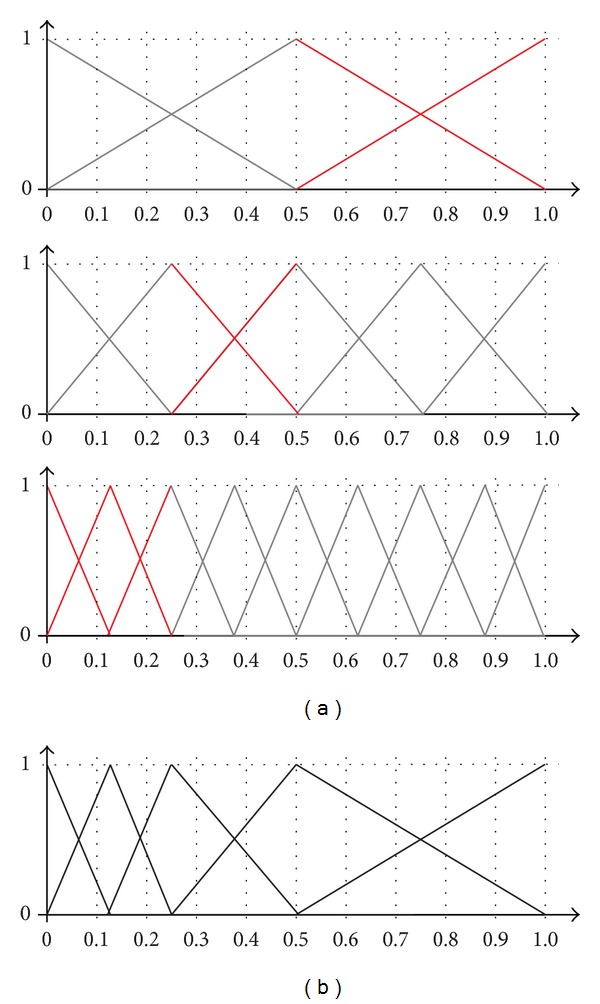
Unbalanced term set with 5 linguistic labels (b) obtained from a linguistic hierarchy of 3 levels (a).

The semantics of each linguistic label is usually given by a trapezoidal or triangular membership function *μ* : *X* → [0,1] that is represented with a tuple *P* = (*p*_1_,  *p*_2_, *p*_3_,  *p*_4_), where *p*_1_ ≤ *p*_2_ ≤ *p*_3_ ≤ *p*_4_ are the points in the reference domain *X* which define the trapezoid. Some special cases can be defined. If *p*_1_ = *p*_2_ and *p*_3_ = *p*_4_, then *P* corresponds to a crisp interval. If *p*_2_ = *p*_3_ the fuzzy set *P* is triangular; otherwise, it is a trapezoid. If *p*_1_ = *p*_2_ = *p*_3_ = *p*_4_, then *P* is called a crisp real number. In most of the applications that use linguistic information, the fuzzy sets associated with each label are equal, symmetric, and uniformly distributed throughout the domain (e.g., the term set (a) in Figure 2). This kind of representation makes it easier to manage and understand the meaning of each label and the mathematical operations carried out over them. For instance, the LOWA aggregation operator takes advantage of this simplicity to consider only the order in which the labels appear in the domain, which means that it can operate in a symbolic way [20] (see Section 2.2).

However, many daily situations involve term sets in which the fuzzy sets are not symmetric or are not distributed uniformly across the domain [16, 36]. For instance, when a student is evaluated, there is usually only one negative term (failed) but several positive terms (pass, good, very good, and excellent). If a control system is analysing the value of a sensor, there is normally a range of terms that can be used within the standard interval of values, but above a certain threshold there is only one term (alarm). In general, the designers of a fuzzy system may be more interested in defining a certain interval of the domain more precisely than other parts, leading to the use of more labels in that interval. The development of unbalanced linguistic label sets and operators to aggregate these kinds of labels has been highlighted as an important area of research [13].

There have been few studies proposing the use of unbalanced sets of terms. Herrera et al. proposed [14, 36] some aggregation operators for linguistic data represented in fuzzy unbalanced linguistic term sets defined using the 2-tuple model [12]. Terms are represented by a pair (*s*, *α*), where *s* is the linguistic label and *α* is a number that represents the translation of symbols into a real scale. This model is able to deal with unbalanced linguistic variables by means of hierarchical linguistic contexts. A linguistic hierarchy [37] is a set of levels, where each level is a balanced linguistic term set with a granularity different from that of the remaining levels in the hierarchy. Each level belonging to a linguistic hierarchy is denoted as *l*(*t*, *n*(*t*)), where *t* is the number that indicates the level of the hierarchy and *n*(*t*) indicates the granularity of the linguistic term set of that level. To build a linguistic hierarchy, the authors propose the fact that the linguistic set of terms for level *t* + 1 is obtained from its predecessor using the expression *L*(*t*, *n*(*t*)) → *L*(*t* + 1,2 · *n*(*t*) − 1).

In order to work with terms from different levels of the hierarchy it is necessary to use transformation functions to translate linguistic terms from one level to another. Considering that LH = *U*_*t*_ *l*(*t*, *n*(*t*)) is a linguistic hierarchy whose linguistic term sets are denoted as *S* ^*n*(*t*)^ =  {*s*_0_^*n*(*t*)^,…, *s*_*n*(*t*)−1_^*n*(*t*)^}, a transformation function from a linguistic label in level *t* to a label in level *t*′ is defined [37] as

(8) $$\begin{array}{c} \text{T}{\text{F}}_{t^{\prime }}^{t}\left(s_{i}^{n(t)},\alpha^{n(t)}\right)=\Delta \left(\frac{\Delta^{-1}\left(s_{i}^{n(t)},\alpha^{n(t)}\right)\cdot \left(n\left(t^{\prime }\right)-1\right)}{n\left(t\right)-1}\right), \end{array}$$

where Δ(*β*) = (*s*_round(*β*)_, *β*-round(*β*)) and Δ^−1^(*s*_*i*_, *α*) = *i* + *α*.

Herrera-Viedma et al. [36] define the LOWA_un_ and ILOWA_un_ operators on the basis of these transformation functions to make an aggregation (and an induced aggregation) of unbalanced linguistic terms.

To give an illustrative example, Figure 2(a) shows a 3-level linguistic hierarchy. The parts in red are used to construct the unbalanced term set depicted in Figure 2(b).

As can be seen in this example, the set of labels used in each of the levels of the hierarchy is balanced and uniformly distributed but, by taking some pieces of each level and putting them together, it is possible to model an unbalanced term set. The main drawbacks to this approach are that it is quite complex to define an appropriate set of labels and the number of levels (and labels) to consider can be quite large. In the example shown above, there are 17 labels in the 3 levels of the hierarchy, whereas the unbalanced term set to be modelled only has 5 labels. Moreover, the definition of the fuzzy sets in each of the levels is very strict, so it is not easy to model an arbitrary set of unbalanced terms. In contrast, as will be mentioned in the rest of the paper, in our proposal the labels can be represented either with trapezoidal or with triangular fuzzy sets with the sole requirement that they define a fuzzy partition.

Another prominent proposal for modelling unbalanced term sets is given by Xu [16]. He argues that when defining an unbalanced term set, the absolute value of the deviation between the indices of two adjoining linguistic labels should increase as the indices of the linguistic labels steadily increase (the term in the centre has index 0, so there are terms with positive and negative indices). Following this idea, he proposes defining a term set with 2*t* − 1 labels in the following way:

(9) St={sβt ∣ β=1−t,23(2−t),24(3−t),  …,0,…,24(t−3),23(t−2),t−1}.

**Figure 3:**
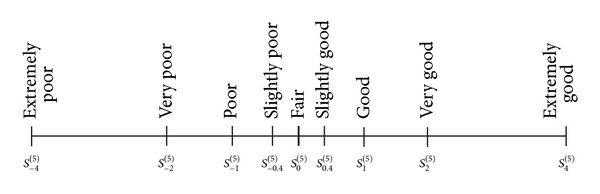
A set of nine linguistic labels (from [16]).

For example, Figure 3 shows an unbalanced term set with nine labels.

It can be seen that this way of defining the unbalanced term sets is very rigid. It is only possible to model those situations in which the labels in the middle are very precise and the labels in the extremes have a wider range. Moreover, the labels are symmetrically located with respect to the centre of the domain, so it is not possible to have more positive labels than negative labels. This strict definition of the term sets allows Xu to define simple functions that permit terms in one set to be transformed into terms in another set. These transformations are meant to be used when different experts have used different term sets to evaluate a set of alternatives. Each label is basically represented by a point in the domain rather than by a fuzzy set.

One of the main aims of our work was to devise a method for working with any unbalanced set of terms, without any restriction on the definition of the fuzzy set associated with each term (as long as a fuzzy partition is obtained). Xu's proposal [16] does not provide this flexibility, since the term sets are very precisely defined and they have to be symmetrically located with respect to the centre of the domain. Moreover, it only considers situations in which precise labels are required in the centre and imprecise labels in the extremes. The study by Herrera et al. [14, 36] allows some unbalanced sets to be modelled provided that the labels are composed by taking pieces from each level of the hierarchy. In contrast, our approach permits the direct definition of the unbalanced term set that fits better with the needs of the application, choosing any fuzzy set for each label. The price to be paid for this flexibility is that the aggregation operator must operate on fuzzy sets.

A related proposal by Xu [21], with which our approach is compared on Section 6, is based on the idea of representing uncertainty by using intervals of values (e.g., an expert could say that the quality of an attribute of an object is “between good and very good”). Xu proposed to consider a fixed and totally ordered discrete term set, for example, *S* = {*s*_−4_, *s*_−3_,…, *s*_3_, *s*_4_}. An* uncertain linguistic variable s* is defined as an interval [*s*_*a*_, *s*_*b*_], where *a* is lower than or equal to *b*. The wider interval is, the more uncertain is the evaluation it provides.

He defines two basic operations on these intervals:

- *s*_1_ + *s*_2_ = [*s*_*a*_1__, *s*_*b*_1__]+[*s*_*a*_2__, *s*_*b*_2__] = [max⁡{*s*_−4_, min⁡{*s*_*a*_1_+*a*_2__, *s*_4_}}, max⁡{*s*_−4_, min⁡{*s*_*b*_1_+*b*_2__, *s*_4_}}],
- *ks* = *k*[*s*_*a*_, *s*_*b*_] = [*s*_*ka*_, *s*_*kb*_], with *k* between 0 and 1.

Note that all the operations are performed on the indexes of the labels, so it is assumed that all terms are homogeneously distributed through the domain. With these two operations, Xu defines the* induced uncertain linguistic OWA aggregator *(IULOWA) as follows:

(10) Xu-IULOWAw=(〈u1,s1〉,〈u2,s2〉,…,〈un,sn〉)=w1sb1+w2sb2+⋯+wnsbn,

where *W* = (*w*_1_, *w*_2_,…, *w*_*n*_) is the usual weighting vector, *u* is the order-inducing variable, and *s*_*b*_*j*__ is the *s* value of the 〈*u*, *s*〉 pair with the *j*th largest *u*. If two pairs have the same *u* value, their two *s* values are replaced by their average.

As will be shown in the comparison in Section 6, the flexibility of our approach allows it to be used to simulate the uncertainty represented by the intervals in Xu's proposal, by replacing these intervals with appropriately defined unbalanced fuzzy sets.

## 3. The Induced ULOWA Operator

The first part of this section focuses on defining the unbalanced linguistic ordered weight averaging operator (ULOWA) which is later extended to create its order-induced version IULOWA.

### 3.1. Unbalanced LOWA (ULOWA) Operator

The ULOWA operator was defined [17] as an extension of the LOWA operator [20] designed to deal with unbalanced linguistic terms. The ULOWA operator takes the same form as the LOWA, which is as follows.

If *A* = {*a*_1_,…, *a*_*m*_} is the set of labels to aggregate, the ULOWA operator is defined as

(11) ULOWA(a1,…,am)=W·BT=Cm{wk,bk,k=1,…,m}=w1⊗b1⊕(1−w1) ⊗Cm−1{βh,bh,h=2,…,m}.

The difference with LOWA lies in the convex combination of two linguistic terms. Whereas LOWA uses a symbol-based approach, ULOWA operates on fuzzy sets; that is, it takes into account the membership functions of the terms. In this way, we are able to take into account the information given by the fuzzy sets during the aggregation process, obtaining a more precise result. So, when *m* = 2, the convex combination of the two terms, *b*_1_ = *s*_*j*_ and *b*_2_ = *s*_*i*_, with *s*_*j*_, *s*_*i*_ ∈ *S*  (*j* ≥ *i*), is calculated taking into account the membership functions of the labels *s*_*j*_ and *s*_*i*_:

(12) $$\begin{array}{c} C^{2}\left\{w_{i},b_{i},i=1,2\right\}=w_{1}⊗s_{j}⊕\left(1-w_{1}\right)⊗s_{i}=s_{k},\\ \text{where }s_{k}\in S\;\text{with}\;k=\underset{i\leq p\leq j}{\arg\;\max}\left\{\text{Sim}\left(S_{p},\delta \right)\right\}. \end{array}$$

In this expression, *δ* is a crisp number defined as *δ* =  (*x*_*k*_, *x*_*k*_, *x*_*k*_, *x*_*k*_) with *x*_*k*_ = *x*_*s*_i__* + *w*_1_(*x*_*s*_j__* −  *x*_*s*_i__*), where *x*_*s*_i__* is the *x*-component of the centre of gravity (COG) of the fuzzy set associated with the label *s*_*i*_ = (*p*_1_, *p*_2_, *p*_3_, *p*_4_):

(13) $$\begin{array}{c} \text{COG}\left(s_{A}\right)=\{\begin{array}{c} y_{A}^{*}=\{\begin{array}{cc} \frac{1}{6}\left(\frac{p_{3}-p_{2}}{p_{4}-p_{1}}+2\right), & \text{if }{\;p}_{1}\neq p_{4},\\ \frac{1}{2}, & \text{if }p_{1}=p_{4}, \end{array}\\ x_{A}^{*}=\frac{y_{A}^{*}\left(p_{3}+p_{2}\right)+\left(p_{4}+p_{1}\right)\left(1-y_{A}^{*}\right)}{2}. \end{array} \end{array}$$

In this way, the aggregation result *s*_*k*_ is the linguistic term consisting of *s*_*j*_ and *s*_*i*_ with the greatest similarity to the intermediate point *δ*. It is worth noting that the result is one of the terms available in the scale of reference *S*. The similarity between two fuzzy sets, proposed after a thorough study of the literature [17], is calculated as follows:

(14) $$\begin{array}{c} \text{Sim}\left(P,Q\right)=\sqrt[{4}]{\overset{4}{\underset{i=1}{\prod }}\left(2-\left|p_{i}-q_{i}\right|\right)}-1. \end{array}$$

**Figure 4:**
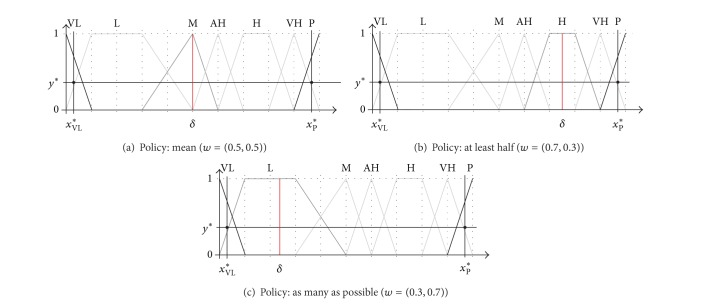
Examples of ULOWA aggregation of two labels (VL and P).

Figure 4 depicts the aggregation procedure of the two extreme labels of an unbalanced 7-term set (VL and P) for different policies. The figure shows the COGs of both labels and their intermediate crisp number *δ* that is used to find the result of the aggregation when varying the vector *W*. Three usual aggregation policies have been used. After the similarity function is applied using (14), ULOWA obtains the neutral term M in the case of* mean*, H in the case of* at least half*, and L in the case of* as many as possible*.

### 3.2. Definition of IULOWA

The induced unbalanced LOWA (IULOWA) is an aggregation operator for linguistic values that are defined on an unbalanced vocabulary *S*. As it is based on IOWA, the operator is able to manage complex decision problems by using order-inducing variables.

**Definition 1 .**

The induced unbalanced linguistic ordered weighted average, based on the ordering criterion *u*, is calculated as

(15) IULOWAw(〈u1,a1〉,〈u2,a2〉,…,〈um,am〉) =W·BT=Cm{wk,bk,k=1,…,m} =w1⊗b1⊕(1−w1)⊗Cm−1{βh,bh,h=2,…,m},

where *B* is the induced ordered vector, that is, *B* = (*a*_*σ*(1)_′, *a*_*σ*(2)_′,…, *a*_*σ*(*j*)_′), where *a*_*σ*(*j*)_′ corresponds to the value *a*_*j*_ having the *j*th largest *u*_*i*_. *W* = (*w*_1_,…, *w*_*m*_) is the usual weighting vector that defines the aggregation policy of the OWA operator, with *w*_*i*_ ∈ [0,1], ∑*w*_*i*_ = 1. The final convex combination of two linguistic terms is the same as in the ULOWA operator defined in (11).

### 3.3. Properties of the IULOWA

As stated in [3], an aggregation operator should have the following properties:* monotonicity*,* commutativity*,* idempotency*, and* boundedness*.

**Property 1 .**

The IULOWA operator is* increasingly monotonous* with respect to the argument values if the associated order-inducing values remain unchanged.

Let us consider two order-induced vectors *P* = {〈*u*_1_, *p*_1_〉,…, 〈*u*_*m*_, *p*_*m*_〉} and *Q* = {〈*u*_1_′, *q*_1_〉,…, 〈*u*_*m*_′, *q*_*m*_〉}, so that ∀*j*, *u*_*j*_ = *u*_*j*_′ and ∀*j*, *p*_*j*_ ≥ *q*_*j*_; then IULOWA_*w*_(*P*) ≥ IULOWA_*w*_(*Q*).

That means, as will be detailed in Section 4, if we replace each term with another one that has the same specificity and fuzziness but a greater preference in the scale *S*, the result will also be an equal or better term in the preference scale. In fact, this case reduces the proof to the ULOWA operator [17], because the inducing variable does not change the order.

**Proof**

Let IULOWA_*w*_(*P*) = IULOWA_*w*_(〈*u*_1_, *p*_1_〉,…, 〈*u*_*m*_, *p*_*m*_〉), and IULOWA_*w*_(*Q*)  =  IULOWA_*w*_(〈*u*_1_′, *q*_1_〉,…, 〈*u*_*m*_′, *q*_*m*_〉). If ∀*j*, *u*_*j*_ = *u*_*j*_′ and ∀*j*, *p*_*j*_ ≥ *q*_*j*_, any induced permutation of the elements satisfies the condition ∀*j*, *p*_*σ*(*j*)_′ ≥ *q*_*σ*(*j*)_′, and IULOWA_*w*_(*p*_*σ*(1)_′,…, *p*_*σ*(*m*)_′) ≥ IULOWA_*w*_(*q*_*σ*(1)_′,…, *q*_*σ*(*m*)_′), due to the monotonicity of the ULOWA operator. Then IULOWA_*w*_(*P*) ≥ IULOWA_*w*_(*Q*).

**Property 2 .**

The IULOWA operator is* commutative*.

IULOWA_*w*_(〈*u*_1_, *a*_1_〉,…, 〈*u*_*m*_, *a*_*m*_〉) = IULOWA_*w*_(〈*u*_1_′, *a*_1_′〉,…, 〈*u*_*m*_′, *a*_*m*_′〉),  where (〈*u*_1_′, *a*_1_′〉,…, 〈*u*_*m*_′, *a*_*m*_′〉) is any permutation of the elements in (〈*u*_1_, *a*_1_〉,…, 〈*u*_*m*_, *a*_*m*_〉).

**Proof**

The IULOWA operator reorders the arguments according to the order-inducing variable. Thus, if *A* = (〈*u*_1_, *a*_1_〉,…, 〈*u*_*m*_, *a*_*m*_〉) is any permutation of *A*′ = (〈*u*_1_′, *a*_1_′〉,…, 〈*u*_*m*_′, *a*_*m*_′〉), the order induced for *A* and *A*′ will be the same. Therefore, IULOWA_*w*_(*A*) = IULOWA_*w*_(*A*′).

**Property 3 .**

The IULOWA operator is* idempotent *in the sense that IULOWA_*w*_(〈*u*_1_, *a*_1_〉,…, 〈*u*_*m*_, *a*_*m*_〉) = *a*, if ∀*j*, *a*_*j*_ = *a*.

**Proof**

The proof does not depend on the inducing variable, because in this case the values to be aggregated are the same for all the arguments. Then, in line with the definition of the IULOWA operator, we have a final step (12) that consists of IULOWA_*w*_(*a*_*m*−1_, *a*_*m*_) = *s*_*k*_, where *k* = *w*_1_ ⊗ *s*_*j*_ ⊕ (1 − *w*_1_) ⊗ *s*_*i*_ = arg max_*i*≤*p*≤*j*_{Sim(*s*_*p*_, *δ*)}. In this case, *i* = *j*, so *s*_*k*_ = *s*_*i*_ = *s*_*j*_ = *a*. Recursively, we obtain IULOWA_*w*_(*a*_1_,…, *a*_*m*_) = *a*.

**Property 4 .**

The IULOWA operator is* bounded. *That is, for any weighting vector *W*,

(16) min⁡(a1,…,am)≤IULOWAw(u1,a1,…,um,am)≤max⁡(a1,…,am).

**Proof**

Given that *s*_*j*_, *s*_*i*_ ∈ *S*  (*j* ≥ *i*), we have defined the convex combination of these two terms as *C* ^2^{*w*_*i*_, *b*_*i*_, *i* = 1,2} = *w*_1_ ⊗ *s*_*j*_ ⊕ (1 − *w*_1_) ⊗ *s*_*i*_ = *s*_*k*_. According to (12), we have *k* = arg max_*i*≤*p*≤*j*_{Sim(*s*_*p*_, *δ*)}. That is, the resulting label from the combination of two labels is *C* ^2^(*s*_*i*_, *s*_*j*_) = *s*_*k*_ with *i* ≤ *k* ≤ *j*. This means that we cannot obtain a result out of the limits given by the labels that are aggregated at each step.

### 3.4. Families of IULOWA Operators

The IULOWA operator permits the definition of a wide range of families of unbalanced linguistic aggregation operators following the methodology used in the OWA literature [6, 21]. Note that each specific case is useful in certain situations depending on the objectives of the analysis. For example, when aggregating *m* labels, we can study the following cases.

- If *w*_*j*_ = 1/*m*, for all *j*, we get the unbalanced linguistic average (ULA).
- The induced unbalanced linguistic maximum is obtained if *w*_1_ = 1 and *w*_*j*_ = 0, for all  *j* ≠ 1, which gives as result the value *a*_*i*_ with maximum *u*_*i*_, because *u*_1_ = max⁡{*u*_*i*_}, after the reordering stage.
- The induced unbalanced linguistic minimum is obtained if *w*_*m*_ = 1 and *w*_*j*_ = 0, for all *j* ≠ *m*, which gives as result the value *a*_*i*_ with minimum *u*_*i*_, because *u*_*m*_ = min⁡{*u*_*i*_}, after the reordering stage.
- The unbalanced linguistic weighted average (ULWA) appears if *u*_*i*_ > *u*_*i*+1_, for all *i*.
- The unbalanced LOWA operator is obtained if the *j*th largest label, *s*_*j*_, according to the scale *S*, is also ordered at position *j* according to the inducing variable *U*, for all *j*.
- Step-IULOWA: it occurs if there is a position 1 ≤ *k* ≤ *m* so that *w*_*k*_ = 1 and *w*_*j*_ = 0, for all *j* ≠ *k*.
- Median-IULOWA: if *m* is odd, we assign *w*_*p*_ = 1 and *w*_*j*_ = 0 for all others, with *p* the position of the [(*m* + 1)/2]th largest *u*_*i*_. If *m* is even, we assign, for example, *w*_*p*_ = *w*_*q*_ = 0.5 and *w*_*j*_ = 0 for all others, with *p* and *q* being the positions of the (*m*/2)th and [(*m*/2) + 1]th largest *u*_*i*_.
- Olympic-IULOWA: it occurs if *w*_*p*_ = *w*_*q*_ = 0, with *u*_*p*_ → max⁡{*u*_*i*_} and *u*_*q*_ → min⁡{*u*_*i*_}, and for all others *w*_*j*_ = 1/(*m* − 2).
- Window-IULOWA: it occurs if *w*_*j*_ = 1/*d* for *k* ≤ *j* ≤ *k* + *d* − 1 and *w*_*j*_ = 0 for *j* > *k* + *d* and *j* < *k*. Note that *k* and *d* must be positive integers so that *k* + *d* − 1 ≤ *m*.
- Centred-IULOWA: it occurs if the aggregation is symmetric, strongly decaying, and inclusive. It is symmetric if *w*_*j*_ = *w*_*m*−*j*+1_. It is strongly decaying when *i* < *j* ≤ (*m* + 1)/2; then *w*_*i*_ < *w*_*j*_ and when *i* > *j* ≥ (*m* + 1)/2; then *w*_*i*_ < *w*_*j*_. It is inclusive if *w*_*j*_ > 0 for all *j*.
- Slide-IULOWA: three versions of this operator can be defined on the basis of the degrees of andness (*α*) and orness (*β*), where *α*, *β* ∈ [0,1] and *α* + *β* ≤ 1, as follows.
  1. Generalised slide-IULOWA occurs when *w*_1_ = (1 − (*α* + *β*))/*m* + *β*, *w*_*m*_ = (1 − (*α* + *β*))/*m* + *α* when and *w*_*j*_ = (1 − (*α* + *β*))/*m*.
  2. Orlike slide-IULOWA occurs if *α* = 0.
  3. Andlike slide-IULOWA occurs if *β* = 0.

## 4. Order-Inducing Variables

In this section we analyse the feature of the IULOWA operator that distinguishes it from the ULOWA operator, which is the order-inducing variable used in the reordering process of the linguistic labels. With this type of operator, we are able to deal with complex reordering processes in which the highest linguistic value in *S* is not the optimal value for the decision maker.

### 4.1. Order Induction

As pointed out in Section 2.1, the order-inducing variable can be obtained using different procedures. The decision maker can express his/her personal ordering directly on the values of the domain of reference, but it is also interesting to have automatic processes to generate the order-inducing criterion. In this latter case the order is linked to certain features of the set of arguments, such as the distance among the values, the past history of values, or the confidence in the values.

In this paper we propose a new way of inducing the order that is related to the additional information given by the shape of unbalanced terms. As mentioned in the introduction, unbalanced terms permit the definition of linguistic variables with different granularity and distribution for the positive and the negative values.

Considering the distribution of the terms {VL, L, M, AH, H, VH, P} in Figure 4, let us assume that we are going to use them to evaluate the performance of a certain object. People usually do not assign extreme values unless they are really sure about the performance of the object; thus, we have defined two very precise fuzzy sets for VL and P (the most negative and most positive terms). Being interested in finding objects with a good performance, there are three terms to indicate different degrees of positivity, while only one indicates a low performance (L). Therefore, L is much more uncertain than the others. Similarly, one can consider that the labels AH (almost high) and VH (very high) are qualifying the term H (high), indicating “a little more than high” or “a bit less than high,” respectively. Thus, they are more precise than high. These specific semantics of the different labels can only be captured using an unbalanced set of terms.

The difference on the certainty of the terms should be taken into account during the aggregation process, as each label is providing a different amount of information about the evaluated alternative. In fact, if we consider that both triangular and trapezoidal fuzzy sets can be associated with the labels (as in Figure 4), then the uncertainty of the labels is not only related to their support intervals in the reference domain but also related to their kernel (i.e., the set of points with value 1).

Taking into account the different features of the definition of the linguistic variables pointed out before, we propose using a measure of the uncertainty of the linguistic labels as the order-inducing criterion for the aggregation. Thus, the arguments will be ordered by decreasing uncertainty. In this way, the contribution of precise labels is prioritized while the effect of uncertain labels is reduced.

In the literature [7, 38–40], two types of uncertainty in fuzzy sets are recognized: (1)* specificity*, related to the measurement of imprecision, which is based on the cardinality of the set, and (2)* fuzziness* or entropy, which measures the vagueness of the set as a result of having imprecise boundaries.

With regard to the measure of* specificity* [8], let *X* be a set and let [0,1]^*X*^ be the class of fuzzy sets on *X*. A measure of specificity is a function Sp: [0,1]^*X*^ → [0,1] so that

1. Sp(*Ø*) = 0;
2. Sp(*μ*) = 1 if and only if *μ* is a singleton;
3. if *μ* and *γ* are normal fuzzy sets in *X* and *μ* ⊂ *γ*, then Sp(*μ*) ≥ Sp(*γ*).

The following specificity measure, for a fuzzy set *A* defined on *X*, is defined as a generalization of other previous formulations [8]:

(17) $$\begin{array}{c} \text{Sp}\left(A\right)=T\left(\alpha_{\sup},N\left(\overset{\alpha_{\sup}}{\underset{0}{\int }}M\left(A_{\alpha }\right)d\alpha \right)\right). \end{array}$$

In this expression *T* is a *T*-norm, ∫_0_^*α*_sup⁡_^ is a Choquet integral, *α*_sup⁡_ is the superior *α*-cut, *N* is a negation operator, and *M* is a fuzzy measure.

A special case of (17) is given in (18), by considering the *T*-norm min, the standard negation *N*(*x*) = 1 − *x*, and the Lebesgue-Stieltjes fuzzy measure *M*([*a*, *b*]) = *b* − *a*. Taking these parameters and a normalized fuzzy set (with *α*_sup⁡_ = 1), the specificity of a fuzzy set defined in the [*a*, *b*] interval can be calculated as

(18) $$\begin{array}{c} \text{Sp}\left(A\right)=1-\frac{\text{area}\;\;\text{under}\;\;A}{b-a}. \end{array}$$

With respect to the measure of* fuzziness* [41], let *X* be a set and let [0,1]^*x*^ be the class of fuzzy sets on *X*. A measure of fuzziness is a function Fz : [0,1]^*x*^ → [0,1] such that

1. Fz(*A*) = 0 if *A* is a crisp set;
2. Fz(*A*) = 1 if ∀*x* ∈ *X*, *A*(*x*) = 1/2;
3. Fz(*A*) ≤ Fz(*B*) if *A* is less fuzzy than *B*; that is, *A*(*x*) ≤ *B*(*x*) ≤ 1/2 or *A*(*x*) ≥ *B*(*x*) ≥ 1/2 for every *x* ∈ *X*.

Fuzziness may be seen as the lack of distinction between the fuzzy set *A* and its complement *A* ^*C*^. A general definition of this type of fuzziness measure is based on an aggregation operator *h* and a distance function *d*, as follows:

(19) $$\begin{array}{c} \text{Fz}\left(A\right)=h_{x\in A}\left(d\left(A\left(X\right),A^{C}\left(x\right)\right)\right). \end{array}$$

For the case of continuous domains using the standard negation operation and the Hamming distance, (19) corresponds to

(20) $$\begin{array}{c} \text{Fz}\left(A\right)=1-\frac{1}{b-a}\overset{b}{\underset{a}{\int }}\left|2\cdot A\left(x\right)-1\right|. \end{array}$$

Specificity and fuzziness refer to two different characteristics of fuzzy sets. Specificity (or its counterpart, nonspecificity [42]) measures the degree of truth of the sentence “containing just one element.” Fuzziness measures the difference from a crisp set. For decision making purposes, it seems desirable to have labels that correspond to single elements rather than to large sets of values, which may hamper the selection of the appropriate alternative. For this reason, we propose using specificity as the order-inducing variable in the aggregation of linguistic terms that qualify a set of alternatives in a decision making process.

**Figure 5:**
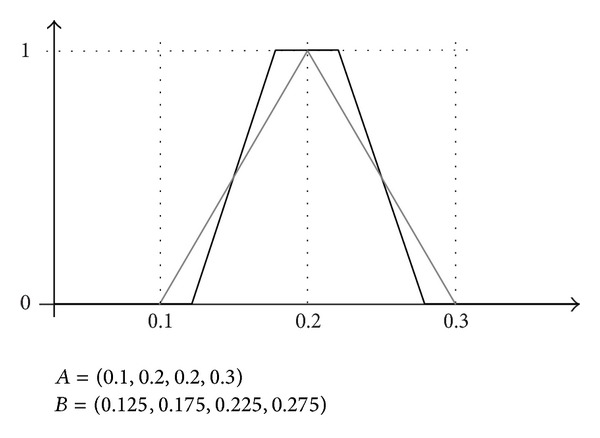
Two fuzzy sets with the same specificity and different fuzziness.

If there are ties between terms with the same specificity, a second ordering criterion may be their fuzziness. An increasing ordering of fuzziness will be used, as we prefer those terms with less uncertainty. If this second criterion also leads to some ties, a decreasing ordering on the preference scale *S* associated with the terms can be used. In Figure 5 we show two fuzzy sets with the same specificity (Sp(*A*) = Sp(*B*) = 0.9), according to (18),

(21) $$\begin{array}{c} \text{Sp}\left(A\right)=1-\frac{\text{area}\;\;\text{of}\;\;A}{b-a}=1-\frac{\left(0.2*1\right)/2}{1-0}=0.9,\\ \text{Sp}\left(B\right)=1-\frac{\text{area}\;\;\text{of}\;\;B}{b-a}=1-\frac{2\left(0.05/2\right)+0.05}{1-0}=0.9, \end{array}$$

but different fuzziness (Fz(*A*) = 0.1 and Fz(*B*) = 0.05), according to (20),

(22) Fz(A)=1−1b−a∫ab|2·A(x)−1|=1−∫01|2·A(x)−1|=1−0.9=0.1,Fz(B)=1−1b−a∫ab|2·B(x)−1|=1−∫01|2·B(x)−1|=1−0.95=0.05.

In this example, the set *A* is fuzzier than *B*, so *B* is preferred.

**Definition 2 .**

Precision-based IULOWA: given a set of unbalanced linguistic arguments {*a*_1_,…, *a*_*m*_} we calculate their induced aggregation according to their uncertainty by using the IULOWA equation (15), where *B* is the induced ordering vector, so that *B* = (*b*_1_, *b*_2_,…, *b*_*m*_) satisfies these conditions:

- ∀*k*1 ≤ *k* < *m* Sp(*b*_*k*_) ≥ Sp(*b*_*k*_ + 1);
- ∀*k*1 ≤ *k* < *m*  if  Sp(*b*_*k*_) = Sp(*b*_*k*_ + 1), then Fz(*b*_*k*_) ≤ Fz(*b*_*k*_ + 1);
- ∀*k*1 ≤ *k* < *m*  if  Sp(*b*_*k*_) = Sp(*b*_*k*_ + 1) and Fz(*b*_*k*_) = Fz(*b*_*k*_ + 1), then *b*_*k*_ > *b*_*k*_ + 1 according to the linguistic scale *S*.

Notice that if the terms correspond to crisp numbers, IULOWA is reduced to the OWA operator.

**Figure 6:**
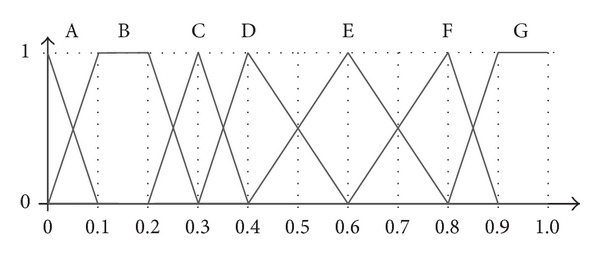
Linguistic variable with 7 terms (test 1).

The following example will show how the terms depicted in Figure 6 would be sorted according to the previous ordering rules.  Table 1 shows the information regarding each of the terms needed to conduct the sorting procedure. Specificity is calculated with (18), whereas fuzziness is obtained with (20).

Taking into account the specificity, the labels are ordered as A > C > (D,  F,  G) > (B, E). Note that there are two ties: the first one between D, F, and G (with Sp = 0.85) and the second one between B and E (Sp = 0.8). Using the fuzziness measure to solve the ties, we put G (Fz = 0.05) before D and F (Fz = 0.15) in the first tie, because we give priority to less fuzzy terms. In the second tie, B (Fz = 0.10) precedes E (Fz = 0.20). As we can see, by measuring fuzziness we are still unable to decide the order between D and F, so we use the index of the terms to decide their position, putting F (index = 5) before D (index = 3). Thus, the induced order according to the procedure proposed in this paper (Definition 2) is A > C > G > F > D > B > E.

### 4.2. Weight Generation

As mentioned above, the OWA weights *w*_*i*_ are used to define different conjunction/disjunction aggregation models [43, 44]. As proposed in the literature [1, 5, 45], the inclusion of an additional variable in the OWA aggregator may also involve the transformation of the set of weights.

In this section we propose modifying the set of weights associated with the arguments by taking into consideration the uncertainty of the values that are aggregated. The rationale is that the more specific values should have a higher weight, whereas the less specific terms (that are less reliable) should have a lower weight.

Using the family of fuzzy quantifiers proposed by Yager [3], the set of weights is obtained with the expression

(23) $$\begin{array}{c} w_{k}=Q\left(\frac{S\left(k\right)}{S\left(n\right)}\right)-Q\left(\frac{S\left(k-1\right)}{S\left(n\right)}\right), \end{array}$$

where *S*(*k*) = ∑_*l*=1_^*k*^ *u*_*σ*(*l*)_ and *σ* is the permutation according to the order-inducing procedure established before. *Q*(*p*) indicates the degree of compatibility of *p* with the concept denoted by *Q*. For example, if *Q* represents a linguistic quantifier such as “most of” and *Q*(0.95) = 1, then it can be said that a value of 95% is completely compatible with the idea conveyed by this linguistic quantifier.

The properties of the quantifier function must be taken into account in order to generate a coherent set of weights for the OWA operator. Taking the usual quantifier *Q*(*r*) = *r* ^*a*^ [3], if *a* ∈ [0,1], then the weighting function is concave, which ensures that the larger the specificity, the higher the weight *w*_*k*_ of the corresponding argument [45]. It is worth noting that with *a* ∈ [0,1] the aggregation policy is disjunctive, which means that uncertain evaluations can be replaced with the most specific available values.

Table 2 shows an example of weights obtained without taking into account the specificities. We have made several tests with different values of the parameter *a*, ranging from 0.1 (where we mostly base the result on the first argument) to 1 (which corresponds to an arithmetic average of the arguments, as the weights are equal for all the values).

To evaluate the impact of the specificity measure in the set of weights, two tests have been performed. The first one is based on the linguistic variable with 7 terms represented in Figure 6. We generate the weights for the values (A, C, F, B, and B) with specificities (0.95,0.9,0.85,0.8,  and  0.8), respectively (see Table 1). The results are shown in Table 3.

In this test, the specificities of the terms that are aggregated are very similar. For this reason, the weights in Table 3 are quite similar to those in Table 2, where specificity was not considered. This shows that when the specificity of the terms is similar, the weights are not heavily modified.

**Figure 7:**
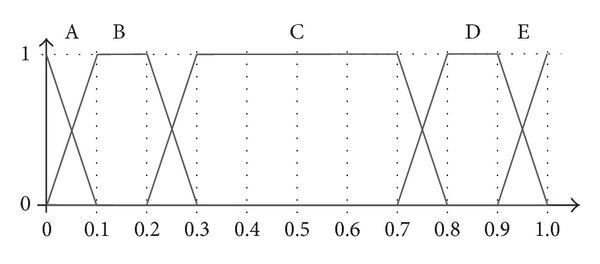
Linguistic variable with 5 terms (Test 2).

For the second test we have used another set of terms with different degrees of specificity, shown in Figure 7. In this case, we aggregate the values (E, B, B, C, and C) with specificities (0.95, 0.8, 0.8, 0.5, and 0.5), respectively.

In this second test the last two terms have a specificity (0.5) much lower than the first three terms (0.95 and 0.8). The results given in Table 4 show that this difference affects the weights as expected, giving more weight to the less uncertain terms. We can see a notable increase in the overall weight of the first three terms and a decrease in the weight of the last two terms.

## 5. IULOWA Multiperson Multicriteria Case Study

In this section we use the operator defined in this paper to address a real environmental evaluation problem. In particular, we study the impact of disposing sewage sludge in agricultural soils.* Environmental impact assessment* is defined by the International Association for Impact Assessment (IAIA) as “the process of identifying, predicting, evaluating and mitigating the biophysical, social, and other relevant effects of development proposals prior to major decisions being taken and commitments made.” In the last decades the increase of sewage sludge production as a residue of wastewater treatment plants (WWTP) has become an environmental problem in several countries. To maintain sustainability, countries are encouraged to promote the value of sewage sludge as a useful by-product. One of the most widespread practices has been to apply sewage sludge to agricultural soils as fertilizer. Although this option is generally accepted because it reduces fertilizer costs, it may have ecological and human impacts. In the SOSTAQUA Spanish research project these impacts have been studied and evaluated using many different criteria. Criteria were structured along three basic axes: economic aspects, environmental suitability, and human health risks [46–48]. For sludge managers, the decision on how to distribute the available sludge (from different WWTPs) among their clients (farmers with different agricultural fields) is quite complex due to the large amount of information that has to be considered and due to the expert knowledge that is required to make a correct evaluation. For this reason, it is important to have tools that evaluate the degree of suitability of using a given sewage sludge on different types of soils in order to find the best possible combination.

In this paper we focus on the problem of obtaining an overall suitability index that evaluates the impact of certain types of sludge on soil. This overall suitability is obtained by aggregating the five criteria presented in Table 5. The evaluation of these criteria is not straightforward and can be made using different methodologies [46, 49]. Moreover, some of the information considered in the evaluation model is subjectively defined by a domain expert, so we can have different opinions from different people.

### 5.1. The Multiperson Multicriteria Aggregation Process

**Figure 8:**
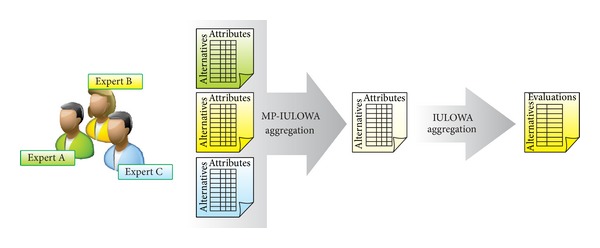
Diagram of the multiperson multicriteria aggregation process.

It is quite common to find problematic decisions when a set of alternatives have been evaluated by different experts on a set of criteria. In this scenario, an aggregation process with two steps is carried out. First, the experts' evaluations of each criterion are fused in order to find a collective result for each criterion. Afterwards, collective criteria are aggregated in order to find the overall evaluation for each alternative. This two-stage process is illustrated in Figure 8.

Let *O* = {*O*_1_, *O*_2_,…, *O*_*n*_} be a finite set of options (or alternatives) to be considered in the group decision making problem. Let *C* = {*C*_1_, *C*_2_,…, *C*_*m*_} be a set of criteria (or attributes). Let *E* = {*E*_1_, *E*_2_,…, *E*_*q*_} be a finite set of experts (or decision makers or stakeholders) who participate in the decision making process, so that each expert *E*_*k*_ provides his/her own payoff matrix (*a*_*ij*_)_*n*×*m*_. The process can be defined as follows.

**Step 1 .**

For each option *O*_*i*_ and each criterion *C*_*j*_, take the *q* values of the experts *E* and calculate the weighting vector *W* to be used in the IULOWA operator, according to the order-inducing variable *U* (i.e., the specificity and fuzziness of the labels), following the method proposed in Section 4. Then apply IULOWA to aggregate the *q* values of the experts *E* using the weighting vector *W*, following Definition 2. The result is the collective payoff matrix (*a*_*ij*_)_*n*×*m*_.

**Step 2 .**

For each option *O*_*i*_ and its collective scores obtained in Step 1, calculate the weighting vector *W* to be used in the IULOWA operator, considering the order-inducing variable *U* (i.e., the specificity and fuzziness of the linguistic labels of the *i*th row of the matrix) and the method proposed in Section 4. Then, calculate the overall aggregated results with the IULOWA operator using Definition 2.

**Step 3 .**

Adopt decisions according to the results found in the previous steps. Select the alternative that provides the best result. Otherwise, establish an ordering or a ranking of the alternatives from the most- to the least-preferred alternative, to enable the consideration of more than one selection.

This double-aggregation process is applied to a given option *O*_*j*_ described with *m* criteria by *q* experts and can be expressed as a function MP-IULOWA : *S* ^*m*^ × *S* ^*q*^ → *S* so that

(24) MP-IULOWA((〈u11,a11〉,…,〈um1,am1〉)…(〈u1q,a1q〉,…,〈umq,amq〉))=IULOWA(IULOWAj=1,…,m×(〈uj1,aj1〉,…,〈ujq,ajq〉)).

Note that, in the literature, there is a wide range of methods for group decision making including those that use expert systems, voting systems, and game theory. Observe that game theory is focused on competitive decision making, where the individuals make a decision considering the potential actions of their opponents. In this context, it is worth noting that the work of Yager [50] considered the use of OWA operator in game theory. This paper has not received much attention in the scientific community but it may bring strong implications for the development of new approaches for group decision making with game theory. For example, all the extensions and generalizations of the OWA operator also have the potential to be implemented under this framework, including the IULOWA operator presented in this paper. By using the IULOWA operator, deeper assumptions should be made because the first step would be to assume that the available information is given in the form of linguistic variables, potentially defined on unbalanced fuzzy sets. Although this point is not considered in this paper, note that this issue may bring some potential developments in future research.

### 5.2. Solving the Case Study

**Figure 9:**
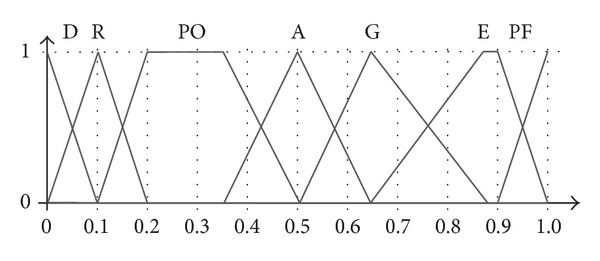
Evaluation scale for the criteria (D: “dangerous,” R: “risky,” PO: “poor,” A: “acceptable,” G: “good,” E: “excellent,” and PF: “perfect”).

In this section we apply the MP-IULOWA operator to an example with 3 types of sludge (*S*1, *S*2, and *S*3) and 4 agricultural fields (*F*1, *F*2, *F*3, and *F*4), which leads to a total of 12 different combinations or cases. Let us assume that three experts (*E*1, *E*2, and *E*3) have evaluated those cases with the five criteria explained in Table 5 and using the unbalanced linguistic variable depicted in Figure 9.

The linguistic vocabulary gives 7 degrees of suitability, ranging from a dangerous to a perfect situation. This linguistic scale presents an unbalanced set of terms with different specificity and fuzziness (see Table 6). The most specific terms are those that correspond to the most extreme scores (dangerous and perfect), followed by the term “risky.” This specificity is needed because those labels refer to very critical and precise situations. A not so specific neutral term is available for use if there is a combination of values that is neither positive nor negative, from the point of view of environmental suitability. The other terms permit the identification of different suitability levels without the need to be too precise.

Notice that, in this vocabulary, the specificity of the terms is a useful indicator to induce the aggregation weights because the most specific values correspond to those terms that are detecting the most interesting situations, from the decision maker's point of view. In fact, the most specific terms give more information than the rest. It is also necessary to take into account the fact that when the specificity of the evaluations is the same, fuzziness is used to solve those ties. The definition, specificity, and fuzziness of the terms depicted in Figure 9 are shown in Table 6. In this example it is assumed that the scientists that provide the evaluations have similar expertise and it is not necessary to assign different confidences to each of them.

Table 7 corresponds to the three experts' evaluations of the twelve cases, taking into account the environmental criteria explained above.

After obtaining the evaluations of the three experts, it is necessary to aggregate all of this information into a single matrix to represent the group opinion regarding the alternatives for the five criteria. As we want to give more confidence to values with high precision, we will use the proposed two-stage IULOWA aggregation process (see Figure 8).

First, when we aggregate the three experts' evaluations to obtain a single evaluation for each attribute of each alternative, we will apply (18) and (20) to give more confidence to the labels with more specificity and less fuzziness.  Table 8 shows the matrix obtained after the aggregation of the three experts' opinions using the IULOWA operator induced by the specificity and by the quantifier *Q*(*r*) = *r* ^0.5^, which corresponds to high* orness*. This policy establishes that the evaluations given by the more uncertain values will be almost ignored, and the overall result will be mostly based on the most specific evaluation given by one of the experts (*w*_1_ around 0.55). For example, for alternative 1 and the “biodiversity” criterion, the ranking of the values given by the three experts is risky ⪰ acceptable ⪰ excellent. The result given by IULOWA is poor and is mainly based on the combination of the two first labels (*w*_1_ + *w*_2_ ≈ 0.85). Thus, as the most precise expert has indicated only a “risky” level of suitability, and taking into account the precise medium evaluation given by “acceptable,” the result is “poor.”

In the resulting matrix, which contains the collective evaluation, IULOWA is applied again to each row in order to obtain a final overall evaluation for each alternative. We follow the same aggregation policy, which weights the contribution of the values according to their precision. The last column of Table 8 shows the overall suitability of each of the twelve alternatives considered.

As indicated above, evaluating the environmental impact of treating soil with sewage sludge is quite a delicate task and the experts will want to give more importance to the cases, where they have detected extreme values such as “dangerous” or “perfect.” Using the IULOWA weighting mechanism and applying a disjunctive aggregation policy, we find that the most specific label contributes around 45% to the final result (*w*_1_ = 0.45). The remaining 55% is divided among the other labels, in particular the one in the second position after the ranking according to the uncertainty. For example, in case 8, the ranking is *acceptable*⪰*excellent*⪰*excellent*⪰*excellent*⪰*good*, so the final result is mainly a combination of “acceptable” and “excellent,” which gives the result “good.”

Notice that, with this criterion, a precise evaluation is considered more important because experts are more confident when they give a specific evaluation that when they choose a more general one. This rationale is clearly exemplified in cases 7 and 9, where an extreme evaluation (positive “perfect” for case 7 and negative “dangerous” for case 9) has direct consequences on the final overall suitability evaluation. In case 7, despite having an “acceptable” evaluation for most of the attributes, the fact of having a single but very specific “perfect” evaluation makes the overall evaluation “good.” A similar event occurs in case 9, where having a “dangerous” evaluation reduces the final evaluation to “poor,” although most of the attributes have an “acceptable” suitability.

## 6. Comparison with Xu-IULOWA Aggregator

**Figure 10:**
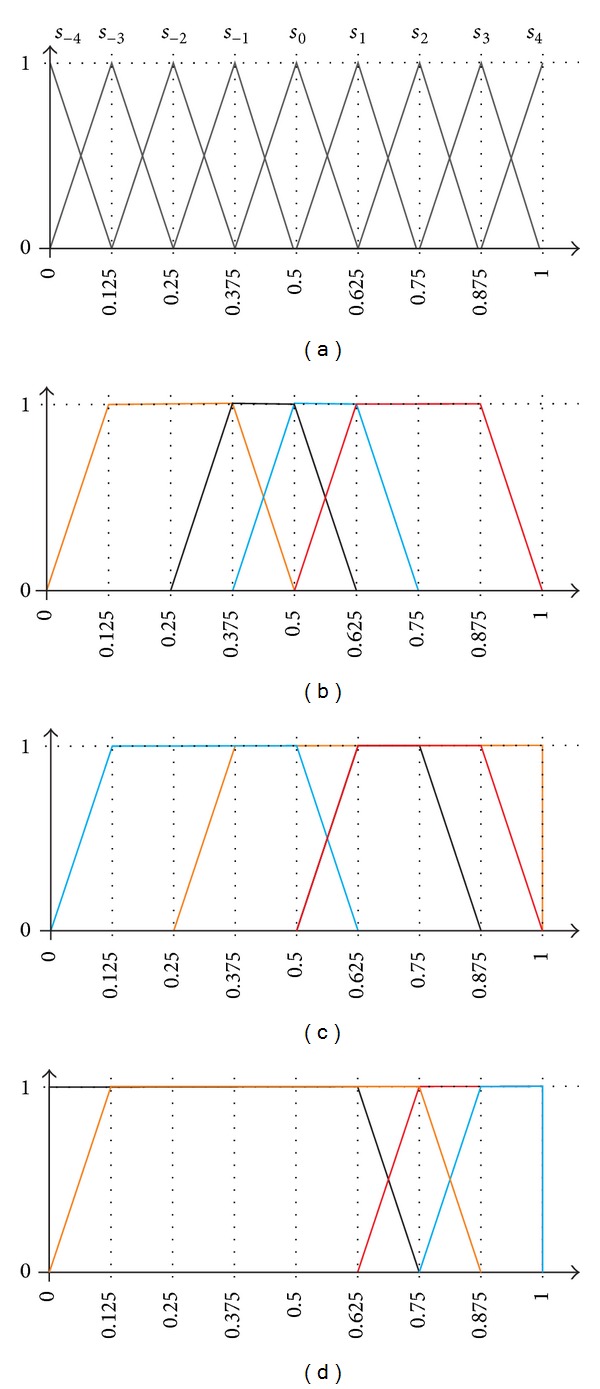
Linguistic terms used in the comparison. (a) Basic set of 9 labels equally distributed; (b) terms aggregated in case 1; (c) terms aggregated in case 2; (d) terms aggregated in case 3.

In this section we compare the performance of the proposed IULOWA aggregator with another well-known induced linguistic operator with the same name defined by Xu [21]. The method proposed by Xu intends to aggregate the information provided by a set of* uncertain linguistic variables*. Each of these variables is defined with an interval of two linguistic terms from a fixed finite set of predefined terms.  Figure 10(a) shows an example set of 9 labels, numbered from *s*_−4_ to *s*_4_, whose meaning may be taken to be implicitly represented by a set of symmetric and uniformly distributed triangular fuzzy sets. Moreover, each item to be aggregated has an associated value that is used to induce the order in which the items have to be aggregated; however, Xu does not propose any specific induction order. In Section 2 (10) we have provided the definition of the Xu-IULOWA operator, which only depends on the indexes of the aggregated elements (no operations are performed explicitly on fuzzy sets). The result of the aggregation is also an uncertain linguistic variable that is an interval [*s*_*a*_, *s*_*b*_], where *a* and *b* do not have to be values from the original set of terms. For example, the result of an aggregation could be [*s*_−1.3_, *s*_2.4_].

In this section we show how our proposal is flexible enough to be able to “simulate” the uncertainty represented by the intervals in Xu's work. The flexibility of our method relies on the possibility of associating any fuzzy set to a label, even if it is unbalanced or the labels are not uniformly distributed throughout the domain of discourse.

Let us consider three case studies. In each of them the aim is to aggregate 4 uncertain linguistic values, as defined by Xu (i.e., 4 intervals of terms defined on a set of 9 labels), using Xu's method and our novel IULOWA operator. In order to apply our method, first it is necessary to translate each interval to a fuzzy set. Our idea is to simulate each interval with a trapezoid, using the triangular fuzzy sets shown in Figure 10(a); for instance, the interval (*s*_−2_, *s*_3_) would be simulated with the trapezoid (0.125, 0.25, 0.875, and 1.0).  Table 9 shows the 4 values to be aggregated in each of the 3 cases and the trapezoids associated with each interval. These trapezoids are also shown in Figures 10(b), 10(c), and 10(d). In order to induce the aggregation order in our method, it is necessary to know the specificity, fuzziness, and index associated with each of the fuzzy sets to be aggregated. The values are shown in Table 9 (the index refers to the relative order of the lowest extreme of the intervals). Having these three values, the order in which the items have to be aggregated is determined (the last column of Table 9). First we take the more specific sets. If two sets have the same specificity, the less fuzzy set is preferred. If two sets have the same specificity and fuzziness, the one with lower index takes precedence.

The next step of our method is to calculate the weighting vector, which depends on the aggregation policy (the parameter *a* described in Section 4.2) and on the specificity of the values to be aggregated (the more specific values have a higher weight than the less specific values).  Table 10 shows the values of the weighting vector for 3 different policies (*a* = 0.1, *a* = 0.5, and *a* = 1) as described on Section 4.2.

After all this process, we can apply our IULOWA aggregator and Xu's IULOWA operator, using in both cases the same induced order and the same weighting vector. The results (a label in our approach, an interval in Xu's case) are also shown in Table 10. It is worth noting that, in our approach, after the aggregation of each pair of labels, we take as result the closest label of the original 9 term set.

The main difference between both proposals is that the result of our method is, by definition, a single label, whereas the result of Xu's approach is an interval. Note that each of the nine original labels may be taken to represent, in some way, a certain interval (e.g., label *s*_1_ corresponds basically to the values between 0.5625 and 0.6875).  Table 10 shows how, in all cases, our result is indeed very close to the middle of the uncertain interval obtained by Xu.

The three cases can be mentioned in more detail. In case 1 (Figure 10(b)) there are two specific labels (−1,0) and (0,1) and two slightly more general ones (1,3) and (−3, −1), defined in a symmetric way with respect to the centre of the domain. When *a* = 1 the two more specific intervals have a slightly higher weight than the others, but the positive ones compensate for the negative ones and the result is perfectly symmetric. When *a* = 0.5 almost 74% of the weight relies on the two initial intervals; although the result is not perfectly symmetric with respect to 0, the first one (−1,0) has a significantly higher weight. When *a* = 0.1 almost all the weight (87.8%) is taken by the first label (−1,0), so the result is very close to this interval.

In case 2 (Figure 10(c)) there are two specific positive labels (1,2) and (1,3) and two labels that are more general (a negative one (−3,0) and a positive one (−1,4)). The difference in the specificity of the labels is clearly reflected on the weights assigned to each label, even when *a* = 1, as the first label is more than twice the weight of the last one (43.3% and 14.4% respectively). This difference is even bigger when *a* = 0.5, where the two initial labels already take 79.2% of the total weight, and it takes its maximum expression when *a* = 0.1 and the first label takes almost 90% of the whole weight. The two initial labels move the result towards the (1,2) interval, although the third label displaces a little bit this interval towards the negative side (especially when *a* = 1 and this third interval still has a weight of 22.8%).

Finally, in case 3 (Figure 10(d)) there are two very positive and specific intervals (3,4) and (2,4) and two very negative and uncertain intervals (−4,1) and (−3,2). The large difference of specificity between the first two intervals and the other two is clearly shown in their associated weights, which give an overall weight to the first two values of 72.7% (*a* = 1), 85.2% (*a* = 0.5), and 96.8% (*a* = 0.1). Thus, the lower is the parameter *a*; the more positive is the result of the aggregation.

In summary, the comparison between our novel method and the one proposed by Xu shows how our IULOWA methodology is flexible enough to model the uncertainty represented by Xu's intervals, giving an overall result that, despite being a single label, closely reflects the middle of the resulting uncertain intervals of Xu-IULOWA.

## 7. Conclusions and Future Work

The fusion of partial evaluations to obtain an overall score for each alternative is usually done with aggregation functions. This paper has presented a new aggregation operator called IULOWA, which enables complex reordering processes to be carried out by using order-inducing variables. In particular, the IOWA operator has been extended to deal with linguistic variables that use unbalanced fuzzy sets. Unbalanced sets of terms allow managing values with different degrees of uncertainty, thus permitting the design of sets of linguistic terms for variables that, due to their nature, require different degrees of precision in different parts of the domain.

First, we have proposed a new procedure to aggregate terms with different degrees of precision. This method is based on the extension principle and it uses operations on the fuzzy sets associated with the linguistic terms that are aggregated. The procedure is recursive, following the well-known LOWA operator.

Second, we have carefully analysed the use of induced variables in unbalanced sets of linguistic terms. The paper has proposed a procedure to use the measurement of uncertainty as an order-inducing criterion in IULOWA. In this approach the decision is based on the less uncertain values. The concept of minimum uncertainty is interpreted as maximum specificity and minimum fuzziness, two well-known measures in fuzzy theory. Ties are solved by taking the linguistic scale of evaluation as the preference degree. The paper also shows that it is useful to modify the weighting policy according to the level of uncertainty to make a coherent aggregation of the values.

It can be clearly seen that we have defined a general operator that includes the ULOWA operator when all the terms have the same specificity and fuzziness. This can also be reduced to the LOWA operator if the terms are balanced. In fact, the IULOWA operator provides a wide range of families of unbalanced linguistic aggregation operators following the methodology used in the OWA literature.

On the basis of the IULOWA operator, a multiperson multicriteria scenario has been presented, proposing a solution to the decision making problem in two steps: (1) using IULOWA to obtain a collective value for each criterion of each alternative and (2) using IULOWA to combine the aggregated values of the different criteria into a single overall evaluation. The final overall linguistic value will identify the best alternative/ alternatives. This model has been used in a real environmental assessment problem, using a set of criteria defined in the Spanish research project SOSTAQUA. The results obtained show that when specific values give more information than the more uncertain ones, the IULOWA operator and the weighting policy proposed in this paper give good and consistent results.

Finally, a thorough comparison with an aggregation operator for uncertain linguistic values defined by Xu [21] has been performed. The versatility of our approach, allowing the definition of unbalanced and not uniformly distributed fuzzy sets to represent the meaning of linguistic labels, permits modelling the uncertain intervals considered by Xu and obtaining results that lie closely within the centre of the intervals obtained with that method.

In future research, we plan to develop further aggregation operators for unbalanced linguistic variables including the possibility of introducing the importance of the variables. This combination of weighting criteria has been already studied in the numerical case so different methodologies can be considered in future research [51]. We are also interested in using this approach for group decision making. We intend to study measures of consensus to evaluate the degree of agreement between the experts and to estimate the proximity between the individual opinion and the collective one [52]. The similarity measure between fuzzy sets that we apply to combine pairs of labels during the aggregation process could also be used for this purpose. As mentioned in the paper, the relationship between this novel method of group decision making and game theory is also worth studying in detail. Finally, this study is part of the Spanish DAMASK research project, in which we are developing a web-based online recommender system. In particular, we will deploy a version of this system that is aimed at helping the user to find holiday destinations and restaurants according to his/her personal preferences [53–55].

## Figures and Tables

**Table 1:** Uncertainty measures for the terms in Figure 6.

Term	Definition	Specificity	Fuzziness	Index
A	(0.0, 0.0, 0.0, 0.1)	0.95	0.05	0
B	(0.0, 0.1, 0.2, 0.3)	0.80	0.10	1
C	(0.2, 0.3, 0.3, 0.4)	0.90	0.10	2
D	(0.3, 0.4, 0.4, 0.6)	0.85	0.15	3
E	(0.4, 0.6, 0.6, 0.8)	0.80	0.20	4
F	(0.6, 0.8, 0.8, 0.9)	0.85	0.15	5
G	(0.8, 0.9, 1.0, 1.0)	0.85	0.05	6

**Table 2:** Weights obtained without specificity.

*a*	Weights
0.1	(0.851, 0.061, 0.038, 0.028, 0.022)
0.25	(0.668, 0.127, 0.085, 0.066, 0.054)
0.5	(0.447, 0.185, 0.142, 0.120, 0.106)
0.75	(0.299, 0.204, 0.179, 0.164, 0.154)
1	(0.200, 0.200, 0.200, 0.200, 0.200)

**Table 3:** Weights obtained in test 1.

*a*	Weights
0.1	(0.860, 0.059, 0.036, 0.025, 0.020)
0.25	(0.686, 0.124, 0.080, 0.060, 0.050)
0.5	(0.470, 0.186, 0.136, 0.110, 0.098)
0.75	(0.322, 0.209, 0.174, 0.152, 0.143)
1	(0.221, 0.209, 0.198, 0.186, 0.186)

**Table 4:** Weights obtained in test 2.

*a*	Weights
0.1	(0.876, 0.056, 0.036, 0.017, 0.015)
0.25	(0.719, 0.119, 0.083, 0.042, 0.037)
0.5	(0.517, 0.187, 0.145, 0.079, 0.072)
0.75	(0.372, 0.216, 0.192, 0.112, 0.108)
1	(0.268, 0.225, 0.225, 0.141, 0.141)

**Table 5:** Environmental criteria.

Criterion name	Description	Information used
Biodiversity suitability	Biodiversity is an indicator of the health of ecosystems. Biodiversity can be adversely affected by metal and organic compound contamination depending on the characteristics of the soil.	Metal concentration in the sludge Organic compounds in the sludge Sludge treatment type Organic matter in the soil Soil texture Soil carbonate level

Nitrates suitability	Contamination of the soil by nutrients should be minimized. Applying sludge containing nitrates to a soil may affect its recommended level of nitrates.	Organic matter in the sludge Sludge treatment type Nitrates available in the sludge Soil texture Nitrates available in the soil

Organic matter suitability	Soil organic matter regulates several processes (e.g., as OM mineralizes slowly, nutrients are released at a slower pace, reducing the potential risk of nitrogen leaching to groundwater).	Organic matter in the sludge Organic matter in the soil Sludge treatment type

pH suitability	Metal contamination in soils is related to its pH. For this reason, basic soils are preferred for sewage sludge treatment. Acid soils should receive sludge with a high pH.	Sludge pH Soil pH

Soil contamination suitability	Soil contamination refers to the presence of heavy metals and organic compounds in a soil. The presence of contaminants in sewage sludge may result in risks to humans and ecosystems. The contaminant's movement between environmental compartments may lead to soil contamination.	Metal concentration in the sludge Organic compounds in the sludge Sludge treatment type Organic matter in the soil Soil texture Soil carbonates level Soil pH

**Table 6:** Definition and values of specificity and fuzziness of the linguistic terms.

Linguistic value		Definition	Specificity	Fuzziness
Dangerous	(D)	(0.0, 0.0, 0.0, 0.1)	0.950	0.050
Risky	(R)	(0.0, 0.1, 0.1, 0.2)	0.900	0.099
Poor	(PO)	(0.1, 0.2, 0.35, 0.5)	0.725	0.125
Acceptable	(A)	(0.35, 0.5, 0.5, 0.65)	0.850	0.150
Good	(G)	(0.5, 0.65, 0.65, 0.875)	0.812	0.187
Excellent	(E)	(0.65, 0.875, 0.9, 1.0)	0.812	0.162
Perfect	(PF)	(0.9, 1.0, 1.0, 1.0)	0.950	0.050

**Table 7:** Evaluations of experts *E*1, *E*2, and *E*3.

Case	Biodiversity			Nutrients suitability			Organic matter suitability			PH suitability			Absence of soil contamination		
	*E*1	*E*2	*E*3	*E*1	*E*2	*E*3	*E*1	*E*2	*E*3	*E*1	*E*2	*E*3	*E*1	*E*2	*E*3
1	A	R	E	D	PO	PO	R	R	R	A	E	G	R	R	D
2	G	G	G	A	G	A	PF	G	PF	PF	PF	PF	PO	PO	D
3	PO	G	A	D	D	D	A	PO	PO	R	R	A	R	R	R
4	A	R	PO	A	PO	A	G	E	G	PF	G	PF	PO	G	PO
5	R	A	D	A	E	A	A	R	A	E	G	PF	G	A	PF
6	PO	G	PO	E	G	G	G	G	E	G	G	A	PO	D	PO
7	G	PO	A	G	G	A	G	PO	G	PF	PF	PF	A	G	E
8	E	E	E	G	A	G	E	E	E	E	E	PF	G	G	G
9	R	D	R	PO	G	PO	A	A	PO	D	D	D	A	A	A
10	A	A	R	G	G	PF	G	G	G	R	D	R	PO	PO	PO
11	G	E	A	G	G	G	E	E	G	G	G	G	G	G	A
12	PO	PO	PO	A	A	PO	A	A	A	PF	PF	PF	PO	PO	PO

**Table 8:** Collective data matrix, including the overall suitability value.

Case	Biodiversity	Nutrients suitability	Organic matter suitability	PH suitability	Absence of soil contamination	Overall suitability
1	Poor	Risky	Risky	Acceptable	Risky	Risky
2	Good	Acceptable	Excellent	Perfect	Risky	Good
3	Acceptable	Dangerous	Acceptable	Risky	Risky	Risky
4	Poor	Acceptable	Excellent	Excellent	Acceptable	Good
5	Risky	Acceptable	Poor	Excellent	Excellent	Acceptable
6	Acceptable	Excellent	Excellent	Acceptable	Risky	Acceptable
7	Acceptable	Acceptable	Acceptable	Perfect	Acceptable	Good
8	Excellent	Acceptable	Excellent	Excellent	Good	Good
9	Risky	Acceptable	Acceptable	Dangerous	Acceptable	Poor
10	Poor	Excellent	Good	Risky	Poor	Acceptable
11	Acceptable	Good	Excellent	Good	Acceptable	Good
12	Poor	Acceptable	Acceptable	Perfect	Poor	Good

**Table 9:** Fuzzy terms and features used during the comparison.

Case	ULOWA pair	Membership function (*p*_1_, *p*_2_, *p*_3_, *p*_4_)	Specificity	Fuzziness	Index	Induced order
1	[*s*_−3_, *s*_−1_]	(0, 0.125, 0.375, 0.5)	0.625	0.125	0	*4 *
	[*s*_0_, *s*_1_]	(0.375, 0.5, 0.625, 0.75)	0.75	0.125	2	*2 *
	[*s*_−1_, *s*_0_]	(0.25, 0.375, 0.5, 0.625)	0.75	0.125	1	*1 *
	[*s*_1_, *s*_3_]	(0.5, 0.625, 0.875, 1)	0.625	0.125	3	*3 *

2	[*s*_1_, *s*_3_]	(0.5, 0.625, 0.875, 1)	0.625	0.125	3	*2 *
	[*s*_−1_, *s*_4_]	(0.25, 0.375, 1, 1)	0.3125	0.062	1	*4 *
	[*s*_−3_, *s*_0_]	(0, 0.125, 0.5, 0.625)	0.5	0.125	0	*3 *
	[*s*_1_, *s*_2_]	(0.5, 0.625, 0.75, 0.875)	0.75	0.125	2	*1 *

3	[*s*_−3_, *s*_2_]	(0, 0.125, 0.75, 0.875)	0.25	0.125	1	*4 *
	[*s*_−4_, *s*_1_]	(0, 0, 0.625, 0.75)	0.3125	0.062	0	*3 *
	[*s*_2_, *s*_4_]	(0.625, 0.75, 1, 1)	0.6875	0.062	2	*2 *
	[*s*_3_, *s*_4_]	(0.75, 0.875, 1, 1)	0.8125	0.062	3	*1 *

**Table 10:** Aggregation of terms.

Case	Terms to aggregate	Policy	Weights	Xu-IULOWA	Our proposal
1	〈(1, [*s*_−1_, *s*_0_]), (2, [*s*_0_, *s*_1_]), (3, [*s*_1_, *s*_3_]), (4, [*s*_−3_, *s*_−1_])〉	*a* = 1	(0.273,0.273,0.227,0.227)	[*s*_−0.72_, *s*_0.72_]	*s*_0_
		*a* = 0.5	(0.523,0.216,0.140,0.121)	[*s*_−0.74_, *s*_0.51_]	*s*_0_
		*a* = 0.1	(0.878,0.063,0.033,0.026)	[*s*_−0.92_, *s*_0.13_]	*s*_0_, almost *s*_−1_

2	〈(1, [*s*_1_, *s*_2_]), (2, [*s*_1_, *s*_3_]), (3, [*s*_−3_, *s*_0_]), (4, [*s*_−1_, *s*_4_])〉	*a* = 1	(0.343,0.285,0.228,0.144)	[*s*_−0.20_, *s*_2.11_]	*s*_1_
		*a* = 0.5	(0.585,0.207,0.133,0.075)	[*s*_0.31_, *s*_2.09_]	*s*_1_
		*a* = 0.1	(0.898,0.056,0.030,0.016)	[*s*_0.84_, *s*_2.02_]	*s*_1_, almost *s*_2_

3	〈(1, [*s*_3_, *s*_4_]), (2, [*s*_2_, *s*_4_]), (3, [*s*_−4_, *s*_1_]), (4, [*s*_−3_, *s*_2_])〉	*a* = 1	(0.394,0.333,0.151,0.122)	[*s*_0.87_, *s*_3.30_]	*s*_2_
		*a* = 0.5	(0.627,0.225,0.084,0.064)	[*s*_1.80_, *s*_3.62_]	*s*_2_, almost *s*_3_
		*a* = 0.1	(0.911,0.057,0.018,0.014)	[*s*_2.73_, *s*_3.91_]	*s*_3_
